# Changes in Glycemic Control Among Individuals With Diabetes Who Used a Personalized Digital Nutrition Platform: Longitudinal Study

**DOI:** 10.2196/32298

**Published:** 2021-10-18

**Authors:** Benjamin Shea, Shivani Bakre, Keaton Carano, Jared Scharen, Jason Langheier, Emily A Hu

**Affiliations:** 1 Foodsmart San Francisco, CA United States; 2 Department of Biostatistics Harvard T.H. Chan School of Public Health Boston, MA United States; 3 Department of Epidemiology Johns Hopkins Bloomberg School of Public Health Baltimore, MD United States

**Keywords:** diabetes, hyperglycemia, hemoglobin A1c, HbA1c, blood glucose, digital health, nutrition, meal planning, food environment, food ordering, food purchasing, platform, longitudinal, characteristic, diet, education

## Abstract

**Background:**

Diabetes-related costs are the highest across all chronic conditions in the United States, with type 2 diabetes accounting for up to 95% of all cases of diabetes. A healthy diet is strongly associated with lowering glycated hemoglobin A_1c_ (HbA_1c_) levels among individuals with diabetes, which can help curtail other health complications. Digital health platforms can offer critical support for improving diet and glycemic control among individuals with diabetes. Less is known about the characteristics of people with diabetes who use digital health platforms (specifically, a platform that integrates personalized healthy meal plans and food ordering) and changes in their HbA_1c_ levels.

**Objective:**

The aim of this study is to characterize Foodsmart users with diabetes and evaluate the longitudinal impact of Foodsmart—a personalized digital nutrition platform with meal planning, food ordering, and nutrition education features—on changes in HbA_1c_ levels.

**Methods:**

We retrospectively analyzed data collected from 643 adults with at least two self-reported HbA_1c_ entries in the Foodsmart platform between January 2016 and June 2021. Participants self-reported their HbA_1c_ levels, height, weight, health conditions, and diet in a 53-item food frequency questionnaire. Diabetes was defined as HbA_1c_ ≥6.5%. We analyzed distributions of characteristics by baseline diabetes status and examined the association of characteristics with the likelihood of having diabetes at baseline. To evaluate the change in HbA_1c_ levels among Foodsmart users, we calculated mean changes (absolute and percent) in HbA_1c_ among participants with diabetes and by length of follow-up. We also compared changes in HbA_1c_ and weight between participants with diabetes at baseline who achieved a normal HbA_1c_ level and those who did not.

**Results:**

We found that 43.5% (280/643) of the participants with at least two HbA_1c_ level entries had diabetes at baseline. Participants with diabetes at baseline were more likely to be male, have a higher weight and BMI, report high blood pressure, and have a poorer diet in comparison to participants without diabetes. Using a multivariable logistic regression model, we found that being male and obese were statistically significantly associated with baseline diabetes. Among participants with diabetes at baseline, HbA_1c_ was reduced, on average, by 0.46%. In addition, 21.4% (60/280) of participants with diabetes achieved a normal HbA_1c_ level (<6.5%) in their last HbA_1c_ level entry; this percentage increased with longer follow-up time (39% [7/18] at >24 months). In a sensitivity analysis, users with an HbA_1c_ ≥7.0% at baseline had an average absolute change of –0.62% and 31.2% (62/199) of these participants achieved HbA_1c_ levels of less than 7.0%.

**Conclusions:**

This study assessed characteristics of individuals enrolled on the Foodsmart platform with HbA_1c_ levels and found that users with diabetes had lower HbA_1c_ levels over time and a sizable percentage of participants were successful in achieving normal levels.

## Introduction

Over 34 million individuals in the United States have diabetes, comprising 13% of US adults [1]. In 2018 alone, 1.8 million new cases were diagnosed [1]. Diabetes-related costs are the highest of any condition in the US health care system, with the cost of care increasing each year [2]. Recent estimates state that direct medical costs of diabetes and related complications amount to approximately $237 billion each year, accounting for one of every seven health care dollars spent [3]. Notably, type 2 diabetes accounts for 90%-95% of all cases in the United States [1].

For adults with diabetes, the body either does not produce enough insulin or its cells are insulin resistant [4]. Because insulin facilitates the uptake of sugar into cells from the bloodstream, diabetes results in elevated levels of blood glucose. Diabetes is defined by high blood sugar, or hyperglycemia, and glycemic control is fundamental to diabetes management [5]. Type 2 diabetes can also occur due to drug-induced hyperglycemia, which is often caused by beta blockers, thiazide diuretics, corticosteroids, and others [6]. In the long-term, high levels of blood glucose can lead to macrovascular (heart disease, stroke, poor blood circulation) and microvascular (loss of sight, nerve damage, and kidney disease) damage [7]; these health issues lead to greater health care costs. By controlling their blood sugar, however, patients can limit the effect of these negative health consequences [7]. A healthy diet is a critical component in this treatment plan; this includes a meal plan of healthy carbohydrates and fats as well as fiber-rich foods, while limiting foods that are high in trans fats, sodium, and added sugars [8]. Generally, healthy diets for those with diabetes or hyperglycemia are nutrient rich and low in fat and calories. The incorporation of this type of diet for those with type 2 diabetes has been shown to decrease individuals’ glycated hemoglobin A_1c_ (HbA_1c_) levels, a measure of one’s mean blood glucose levels over the prior three months [9,10]. Despite evidence of the benefits of a healthy diet, there are many barriers to adopting and sustaining these changes in one’s diet, such as lack of time, financial resources, accessibility, and information [11]. Previous studies have shown that lower income neighborhoods have an increased exposure to advertisements for tobacco and alcohol, and are more likely to be food deserts, with less access to healthy foods [12,13]. A study conducted in the United Kingdom found that while many participants understood what a healthy diet was, they found it difficult to achieve due to lack of time, advertising, community norms, and conflicting advice from professionals [14]. Furthermore, dietary habits are also shaped by education and nutritional and cooking knowledge, as well as motivation and convenience [15].

Foodsmart is a meal planning platform that addresses access to affordable and healthy foods to enable its users to develop healthy eating habits. The Foodsmart platform improves its participants’ health by providing users with a basic understanding of their current diet and potential areas for improvement. It also supplies participants with personalized recipe recommendations and facilitates the purchasing of healthy options through ad-free online ordering of groceries, meal kits, and prepared foods at discounted prices. Previous research has shown that Foodsmart members with obesity have achieved weight loss that has been sustained over the time during which they used the platform [16].

Previous studies have shown that digital nutritional and dietary interventions can improve glycemic control among individuals with type 2 diabetes [17-19]. One digital low-carbohydrate intervention (with comprehensive diabetes and nutritional education and a social support component) was associated with a mean absolute decrease of 1.17% in HbA_1c_ levels after one year [17]. In another study, a dietary intervention that included both a low-carbohydrate Mediterranean diet and a low-fat diet that was conducted over the course of four years showed that changes in one’s diet can lead to sustained differences in HbA_1c_ levels [18]. Another study’s intervention aimed to encourage participants to adopt a plant-based diet and engage in regular exercise through a digital intervention paired with specialized human support, resulting in a mean change in HbA_1c_ of –0.8% within 12 weeks [19]. In a systematic review of internet interventions, the majority of web-based interventions also focused on the glucose monitoring process and on insulin titration, while very few focused on lifestyle modification, behavior theory, and education with tailored feedback [20,21]. Of the 9 studies that fit the review’s criteria and did promote behavior change, 6 of them targeted healthy eating, which further validates the effect of diet on diabetes [21]. Foodsmart differs from these interventions in its complete digital interface, personalization of meal planning, and online food ordering system. The platform alters the food purchasing environment by integrating recipe recommendations into a grocery list, removing online advertisements for unhealthy options, and providing discounts and price comparisons, all of which ease the process of behavior change. By assisting participants through the process of making healthy, sustainable behavior changes, Foodsmart may, in turn, be able to assist users living with diabetes in reducing their HbA_1c_ levels. Given the complexities of healthy eating, especially among people with diabetes, we wanted to characterize users with diabetes who used Foodsmart.

The main objectives of this study were to better understand and characterize participants with diabetes compared to participants without diabetes and evaluate changes in HbA_1c_ levels, weight, and nutrition quality over time among Foodsmart participants with diabetes through its features including nutritional assessment, personalized meal planning, and altered food environment for grocery purchasing.

## Methods

### Study Sample

As of June 2021, 10,197 participants (aged >18 years and living in the United States) of Foodsmart who enrolled since January 2016 had entered a plausible value for HbA_1c_ (HbA_1c_ >3% or HbA_1c_ <15%). Of those, 643 Foodsmart participants had entered at least two HbA_1c_ entries, with the first and last entry at least 30 days apart. The final sample size was 643 participants who had at least two reports of HbA_1c_.

### Foodsmart

Foodsmart is a digital nutrition platform that encourages sustained behavior change through nutrition education and personalized meal planning, and promotes healthy eating and nutrition through online grocery and food ordering integration. Foodsmart has two components, FoodSmart and FoodsMart, to help users learn how to eat healthy to meet their nutrition targets and order affordable, tasty, and healthy food online, respectively.

The FoodSmart component provides participants with digital dietetics information on how to better plan meals to meet their nutrition targets. Once participants enroll, they are prompted to fill out the Nutriquiz, an online dietary assessment. Participants report their usual dietary intake and meal planning habits and based on the responses, the assessment provides specific dietary recommendations and a tailored meal plan. Participants can retake the Nutriquiz assessment at any time to track their progress toward their health goals.

The second component is FoodsMart, an online food purchasing environment that promotes buying healthy groceries and meals. Personalized meal plans are converted into a grocery list and integrated into online ordering and delivery of meal kits, prepared foods, and groceries. Participants are encouraged to purchase healthy options that align with their preferences and personalized meal plan. Customized grocery discounts for healthier food options and budget-based purchasing that compares prices across integrated grocery partners help participants save money and further encourage participants to choose healthy food options.

Foodsmart is available through health plans and employers and can be accessed via the web or iOS or Android operating systems.

### Measurements of HbA_1c_ and Weight

On the Foodsmart platform, participants were able to enter biometrics such as height, weight, HbA_1c_, blood pressure, and lipids, and were able to update their biometrics at any time. Given the potential for error when entering self-reported metrics, the following values were considered as incorrect entries and were replaced with a missing value: HbA_1c_ ≤3% or ≥15%, BMI ≤15 kg/m^2^ or ≥50 kg/m^2^, and weight ≤27.2 kilograms or ≥181.1 kilograms. We only included participants who reported an HbA_1c_ measurement at least twice, and we used the first (baseline) and last (end) values entered. Length of follow-up was calculated as the number of months between the date of the first value and the date of the last value. We defined HbA_1c_ ≥6.5% as the cutoff for diabetes as defined by the American Diabetes Association [22]. The same method was applied to the end HbA_1c_ value to assess diabetes status at the end of follow-up. Since a glycemic target of HbA_1c_ <7% is recommended for nonpregnant adults, as defined by the American Diabetes Association, we used the cutoff of 7% for sensitivity analyses [5]. Changes in HbA_1c_ were calculated by subtracting the first reported value from the end value. Percent change was calculated by dividing the change in HbA_1c_ by the first HbA_1c_ entry.

Baseline BMI was calculated as the first weight entry in kilograms divided by height in meters squared (kg/m^2^). Participants’ baseline BMI was categorized as normal BMI (BMI <25 kg/m^2^), overweight (25-29.9 kg/m^2^), or obese (BMI ≥30 kg/m^2^). Participants were also able to report any conditions they currently had (eg, high blood pressure, high cholesterol) in the Nutriquiz.

### Dietary Assessment

Participants self-reported their usual dietary intake and habits in Foodsmart. Upon enrollment, participants were prompted to fill out a 53-item food frequency questionnaire called Nutriquiz (adapted from the National Cancer Institute Diet History Questionnaire I [23]). Demographic information (age, sex, height), weight, and daily dietary intake (added sugars, fiber, fruits, vegetables, whole grains, fats, proteins, water, and sodium) were also obtained using the Nutriquiz.

Based on responses from the Nutriquiz, a score (Nutriscore) was calculated to assess overall diet quality, which is based on the Alternative Healthy Eating Index-2010 and the Commonwealth Scientific and Industrial Research Organization Healthy Diet Score [24,25]. Participants were assigned a total Nutriscore from 0 to 70 based on the sum of scores for 7 components: fruits, vegetables, protein ratio (white meat/vegetarian protein to red/processed meat), carbohydrate ratio (total fiber to total carbohydrate), fat ratio (polyunsaturated to saturated/trans fats), sodium, and hydration (percent of daily fluid goal). Each of the components was scored from 0 to 10, with 10 being optimal. Change in the Nutriscore was calculated as a participant’s last Nutriscore minus the participant’s first Nutriscore. A positive change in Nutriscore indicates the participant improved their dietary quality.

### Statistical Analysis

We used descriptive analyses to examine the baseline demographic characteristics, HbA_1c_ levels, and diet quality of the study population as a whole and according to whether participants had diabetes at baseline or not. We reported categorical variables as number of participants (percentage of study population) and continuous variables as mean (SD). We used chi-square tests to assess whether categorical variables are independent of baseline diabetes status, and two-sample *t* tests to evaluate differences in continuous variables.

Univariate and multivariable logistic regression were used to estimate the odds ratios (ORs) and 95% CIs of having diabetes at baseline. The multivariable logistic regression model was mutually adjusted for gender, age category, baseline BMI category, baseline Nutriscore, high blood pressure, and high cholesterol.

Among participants who had diabetes, we calculated the mean changes in HbA_1c_ overall and by time of follow-up (>6 months, >12 months, >24 months). We used paired *t* tests to test whether the changes were statistically significant. Additionally, we calculated the mean percent change for HbA_1c_. In a sensitivity analysis, we used a threshold of HbA_1c_ ≥7% to calculate mean changes in HbA_1c_.

We also calculated the percentage of participants with diabetes at baseline who returned to normal HbA_1c_ levels by the end of follow-up, and stratified by follow-up length. We conducted a sensitivity analysis using a threshold of HbA_1c_ ≥7%.

To further explore the performance of HbA_1c_, we examined changes in weight and HbA_1c_ stratified by whether participants with diabetes at baseline achieved normal HbA_1c_ levels (HbA_1c_ ≥6.5%) by the end of follow-up.

We considered *P* values less than .05 to be significant for all tests. R Studio (version 1.4.1106) and R (version 4.0.5; R Foundation for Statistical Computing) were used for all analyses.

The study was declared exempt from institutional review board oversight by the Pearl Institutional Review Board given the retrospective design of the study and the less than minimal risk to participants.

## Results

### Participant Characteristics

Baseline characteristics of the total study sample and those stratified by baseline diabetes status are shown in Table 1. We found that 43.5% (280/643) of participants had diabetes at baseline. There were 643 participants included in the analysis, of which 64% (411/643) were female and 61% (391/643) were between 40 and 59 years old (Table 1). The mean weight was 93.9 (SD 23.8) kilograms, the mean baseline Nutriscore was 31.4 (SD 8.5) points, and the mean change in the Nutriscore was 3.2 (SD 7.1) points. The mean follow-up length was 10.4 (SD 7.1) months and ranged from 1 to 38 months. Compared to participants who did not have diabetes, participants who did have diabetes were significantly more likely to be male, to have a higher weight and BMI, to have a lower baseline Nutriscore, and to self-report having high blood pressure. Participants with diabetes at baseline were also more likely to have a higher increase in Nutriscore, a longer follow-up duration, and self-reported high cholesterol compared with participants without diabetes at baseline, although the differences were not statistically significant.

To better understand what type of participant was likely to have diabetes at baseline, we examined the association between baseline characteristics and odds of having diabetes in univariate and multivariable logistic regression models (Table 2). In the univariate regression models, participants who were female were 40% less likely to have diabetes at baseline than participants who were male (OR 0.60, 95% CI 0.43-0.82, *P*=.002). Participants classified in the overweight BMI category were 86% more likely to have diabetes at baseline than participants classified in the normal BMI category (OR 1.86, 95% CI 1.10-3.19, *P*=.02). Participants classified in the obese BMI category were 151% more likely to have diabetes at baseline than participants classified in the normal BMI category (OR 2.51, 95% CI 1.58-4.09, *P*<.001). Participants who self-reported having high blood pressure were also 46% more likely to have diabetes at baseline than participants who did not self-report having high blood pressure (OR 1.46, 95% CI 1.06-1.99, *P*=.02). Participants with a higher baseline Nutriscore were less likely to have diabetes at baseline (OR 0.98, 95% CI 0.96-1.00, *P*=.03).

After adjusting for all other variables in the multivariable logistic regression model, we found that being female was associated with 44% lower odds of having diabetes at baseline (OR 0.56, 95% CI 0.39-0.79, *P*=.001). Additionally, participants who were obese were 134% more likely to have diabetes at baseline than those in the normal BMI category (OR 2.34, 95% CI 1.40-3.97, *P*=.001).

**Table 1:** Baseline characteristics of total study sample and by baseline diabetes status.

Characteristic		Total participants			Participants without diabetes			Participants with diabetes			*P* value^a^	
		Sample size, n	Values	Sample size, n		Values	Sample size, n		Values			
Female, n (%)		643	411 (64)	363		251 (69)	280		160 (57)	.002		
**Age (years), n (%)**												.65
	<40	643	35 (5)	363		19 (5)	280		16 (6)			
	40-59	643	391 (61)	363		216 (60)	280		175 (63)			
	≥60	643	217 (34)	363		128 (35)	280		89 (32)			
Weight (kg), mean (SD)		637	93.9 (23.8)	362		90.3 (23.5)	275		98.4 (23.4)	<.001		
Change in weight (kg), mean (SD)		466	–1.7 (8.0)	265		–0.8 (8.3)	201		–2.9 (7.6)	.005		
**BMI category, n (%)**												<.001
	Normal	643	106 (17)	363		77 (21)	280		29 (10)			
	Overweight	643	158 (25)	363		93 (26)	280		65 (23)			
	Obese	643	360 (56)	363		185 (51)	280		175 (62)			
	Missing	643	19 (3)	363		8 (2)	280		11 (4)			
Baseline HbA_1c_ (%), mean (SD)		643	6.6 (1.4)	363		5.8 (0.5)	280		7.8 (1.5)	<.001		
Follow-up duration (months), mean (SD)		643	10.4 (7.1)	363		10.1 (7.0)	280		10.7 (7.3)	.34		
High blood pressure, n (%)		147	47	70		44	78		53	.02		
High cholesterol, n (%)		209	57	92		54	120		61	.10		
Baseline Nutriscore (0-70), mean (SD)		643	31.4 (8.5)	363		32 (8.5)	280		30.5 (8.5)	.03		
Change in Nutriscore, mean (SD)		601	3.2 (7.1)	337		3.0 (7.1)	264		3.4 (7.0)	.46		

^a^Chi-square tests and two-sample *t* tests were used to test differences for categorical and continuous variables, respectively.

**Table 2:** Association between baseline characteristics and likelihood of diabetes at baseline in univariate and multivariable logistic regression models.

Parameter			Univariate odds ratio (95% CI)		*P* value		Multivariable odds ratio (95% CI)		*P* value
Gender (female)			0.60 (0.43-0.82)		.002		0.56 (0.39-0.79)		.001
**Age (years)**									
	<40	1 (reference)				1 (reference)			
	40-59	0.96 (0.48-1.95)		.91		0.84 (0.41-1.78)		.65	
	≥60	0.83 (0.40-1.71)		.60		0.72 (0.34-1.57)		.40	
**Baseline BMI category**									
	Normal	1 (reference)				1 (reference)			
	Overweight	1.86 (1.10-3.19)		.02		1.64 (0.96-2.85)		.08	
	Obese	2.51 (1.58-4.09)		<.001		2.34 (1.40-3.97)		.001	
High blood pressure			1.46 (1.06-1.99)		.02		1.18 (0.83-1.69)		.36
High cholesterol			1.32 (0.96-1.81)		.09		1.13 (0.80-1.60)		.48
Baseline Nutriscore (0-70)			0.98 (0.96-1.00)		.03		0.99 (0.97-1.01)		.47

### Changes in HbA_1c_ Levels

**Figure 1:**
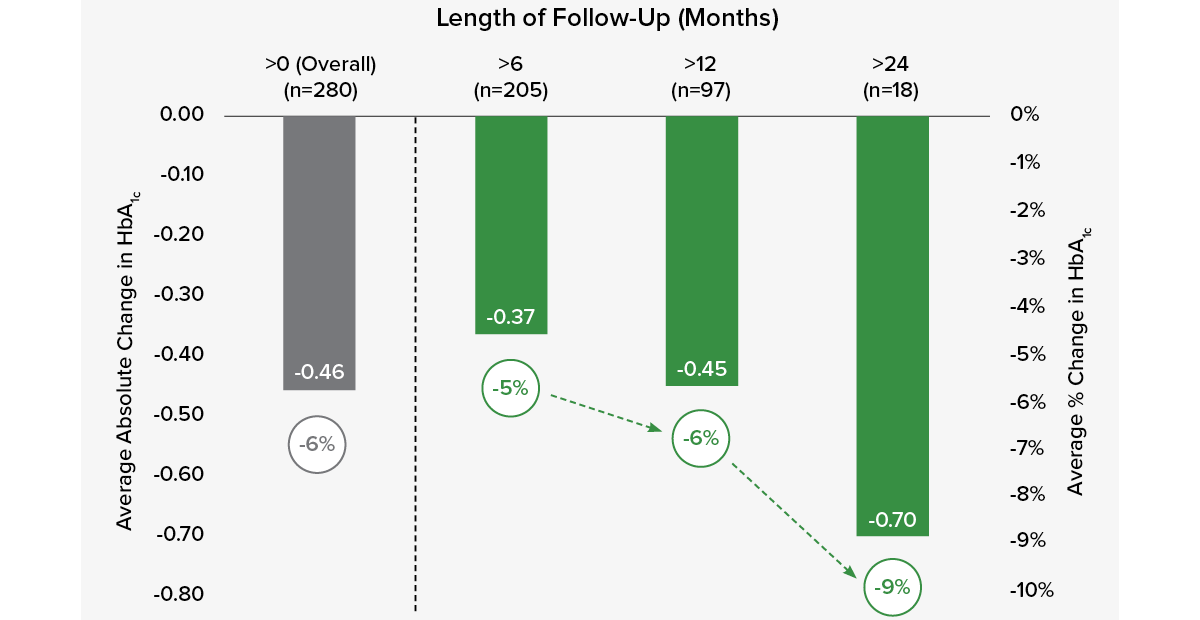
Mean change in HbA_1c_ among participants who had diabetes at baseline. Bars represent average HbA_1c_ change. Line represents average HbA_1c_ percent change.

Figure 1 presents the mean and percent changes in HbA_1c_ levels among participants who were classified as having diabetes for the overall group and by length of follow-up, at >6, >12, and >24 months. The mean changes in HbA_1c_ overall and at >6, >12, and >24 months were –0.46, –0.37, –0.45, and –0.70 points, respectively. Percent changes in HbA_1c_ overall and at >6, >12, and >24 months were –6%, –5%, –6%, and –9%, respectively. All changes were statistically significant (*P*<.05) using paired *t* tests. For users with an HbA_1c_ ≥7.0%, mean change in HbA_1c_ was –0.62 points (*P*<.001), and percent change was –7.6%.

We calculated the percentage of participants with diabetes at baseline who achieved a normal (<6.5%) HbA_1c_ level overall and by cumulative length of follow-up time. Among all participants with diabetes, 21.4% (60/280) achieved normal HbA_1c_ levels, using a threshold of 6.5%. Among participants whose follow-up time was longer than 6, 12, and 24 months, the percentage of participants who achieved normal HbA_1c_ levels was 21.0% (43/205), 22% (21/97), and 39% (7/18), respectively. In a sensitivity analysis, for participants with an HbA_1c_ ≥7%, 31.2% (62/199) of them achieved an HbA_1c_ level less than 7%.

To better understand how weight and HbA_1c_ changed according to end diabetes status, we examined changes in weight and HbA_1c_ stratified by whether participants with diabetes at baseline achieved normal HbA_1c_ levels (Table 3). Reductions in weight and HbA_1c_ were greater for those who achieved normal HbA_1c_ levels at the end of follow-up versus those who did not.

**Table 3:** Change in biometrics stratified by whether participants with diabetes at baseline achieved a normal HbA_1c_ level.

Biometrics	Diabetes to normal	Diabetes to diabetes
Weight change (kg)	–4.1	–2.5
HbA_1c_ change (%)	–1.7	–0.1

## Discussion

### Principal Findings

In this study of 643 participants who used the Foodsmart platform, we found that 43.5% (280/643) had diabetes at baseline, as defined by their baseline HbA_1c_ level. Foodsmart participants with diabetes at baseline were more likely to be male and have a higher weight and BMI. On average, HbA_1c_ decreased by 0.46% among participants with diabetes over a mean duration of follow-up of 10.7 (SD 7.3) months. Among participants with diabetes at baseline, 21.4% (60/280) of those participants achieved a normal HbA_1c_ level by the end of follow-up. These findings suggest that use of the Foodsmart platform may be associated with improved glycemic control among users with diabetes.

In line with our findings, prior studies evaluating the association between diet interventions and clinical biomarkers showed that various nutrition therapies significantly improved glucose regulation and reduced HbA_1c_ levels in patients with diabetes mellitus. For instance, Esposito et al [18] conducted a randomized trial to evaluate the effects of a low-carbohydrate Mediterranean diet versus a low-fat diet on HbA_1c_ levels among individuals with type 2 diabetes. The trial was conducted in Italy and included 215 participants with type 2 diabetes who were classified as obese, had never previously taken antihyperglycemic medication, and had HbA_1c_ less than 11%. After two years, those on the low-carbohydrate Mediterranean diet had a decrease in HbA_1c_ of 1.1%, while those on the low-fat diet had a decrease of 0.5%. In our study, participants who had a follow-up time period greater than 2 years were observed to have a 0.7% decrease in HbA_1c_. Esposito et al [18] also found that, at the end of their study, 37% and 24% of participants returned to normal HbA_1c_ levels (using a threshold of 7%) after following the Mediterranean and low-fat diet, respectively. In our study, 31% of participants returned to normal HbA_1c_ levels (using a threshold of 7.0%). The effects of the low-carbohydrate Mediterranean diet were likely greater than what was observed with Foodsmart because the trial had more specific and stringent guidelines for the participants’ diets than there were with Foodsmart, as participants can make changes that best fit their lifestyles. Therefore, with assistance from Foodsmart in making flexible dietary changes, participants can see changes in their HbA_1c_ levels that are similar to that of a strict low-fat diet.

In our study, we found that men were more likely to have diabetes, which is in line with a previous study conducted by Nordström et al [26]. The authors analyzed data from The Healthy Aging Initiative, a population-based prospective study of men and women 70 years of age or older in northern Sweden, and found a significantly greater prevalence of diabetes in men than women. They hypothesized that this was due to differences in visceral fat mass among men and women and found that when visceral fat mass was adjusted for, male sex was no longer associated with diabetes. Therefore, their findings suggest that differences in the prevalence of diabetes between males and females may be due to differences in visceral fat mass, which is known to be a strong predictor of diabetes [27,28]. Additionally, we found that people with diabetes were more likely to be obese, which has been established by several studies that have found obesity to be a risk factor for diabetes [29-32]. Finally, we found that there was greater weight change among those who achieved normal HbA_1c_ levels. This is consistent with findings from Gummesson et al [33], who conducted a systematic review aimed at understanding the association between weight loss and HbA_1c_ for overweight and obese patients with type 2 diabetes. They found a dose-response relationship between weight loss and reduction in HbA_1c_ in their participants, which may explain why we see a larger weight change for those who have a greater reduction in HbA_1c_ and return to normal levels in our study.

Patients with diagnosed diabetes incur mean medical expenditures of $16,750 per year, of which about $9600 is attributed to diabetes [3]. Glucose-lowering drugs such as sodium–glucose cotransporter 2 (SGLT2) inhibitors and glucagon-like peptide 1 (GLP-1) receptor agonists account for a large proportion of these expenditures, with an estimated mean annual cost of $2727 per patient [34]. Significant savings can be achieved if patients meet the target American Diabetes Association HbA_1c_ level of <7% [5,22]. A 1%, 1.25%, or 1.5% reduction in HbA_1c_ for a commercially insured patient could result in savings of $801, $1033, and $1266 per patient per year, respectively [35]. In this study, participants with diabetes at baseline who achieved an end normal HbA_1c_ level reduced their HbA_1c_ by 1.7% on average, achieving a clinically significant change of 0.5% [36]. Given the high cost of medications, prevention and management of diabetes through eating healthier could be an attractive, low-cost alternative. Unfortunately, we do not know whether participants were on glucose-lowering medications before or during enrollment on the Foodsmart platform. Despite this, we can estimate the difference in costs between prescription medications and Foodsmart. Improved glucose management would cost $327 more per person with diabetes annually relative to current care, largely due to use of antihyperglycemic medications [37]. In comparison, as of 2021, the Foodsmart platform on average costs $12.30 per eligible member annually. Using the results above, a 1% reduction in HbA_1c_ would cost $26.98 on average. On the other hand, using metformin or liraglutide (a GLP-1 receptor agonist) to reduce HbA_1c_ levels by 1% would cost on average $120 and $8640, respectively. Given that the cost of metformin is 4 times higher than the cost of using Foodsmart, and assuming participants on the Foodsmart platform were not on glucose-lowering medication, the cost of a digital platform like Foodsmart would be significantly more affordable than standard treatment with diabetes medications [38-40].

There are some important limitations to note for this study. The first is that HbA_1c_ levels were self-reported and were not clinically validated. However, these values should still be fairly accurate, particularly for participants with diabetes who used the app to track their HbA_1c_ levels. Since participants were not required to enter HbA_1c_ levels, we have reason to believe people who did—in particular, participants with diabetes—had purposefully entered their HbA_1c_ levels rather than entering an arbitrary HbA_1c_ level, which would lead to greater inaccuracy. Additionally, follow-up time was based on when the biometrics were entered, but did not necessarily line up with when the labs were conducted. Another issue is potential selection bias for participants with diabetes who choose to use the platform and are included in the study. For example, those with diabetes who use the app, particularly as a tracker, might be more inclined to want to make changes to their lifestyle. They may have made changes outside of what they did in the app that resulted in changes in HbA_1c_. Therefore, we cannot definitively conclude that Foodsmart's platform caused these changes in HbA_1c_, but there could be an association between using the platform and HbA_1c_ changes. A randomized controlled trial must be conducted to determine if there is a causal link. In addition, there are other potential factors influencing diabetes status at baseline and changes in HbA_1c_ that we might not be able to evaluate because certain types of data are not collected in the Foodsmart app. For example, we do not have participants’ personal or family medical histories to understand their influence on diabetes status [41]. We are also unable to assess how the use of diabetic medications may influence HbA_1c_, as well as other medication-induced fluctuations in HbA_1c_. However, either prevalence of use of these medications or the incident hyperglycemia as a result of these medications in the US population is fairly rare [42]. Some other influencing factors for HbA_1c_ that we did not collect include sleep and amount of exercise [43,44]. We also did not account for socioeconomic factors, such as educational level, which might confound the associations seen and the accuracy of the self-reported biometrics, as stated earlier [45]. Additional studies are required to obtain more information about these covariates. We also did not account for the frequency of use of the Foodsmart platform, which could affect the associations found. Finally, due to missing data for several biometrics (such as BMI) and only single values input for HbA_1c_, our study had a small sample size relative to the total number of participants who use the Foodsmart platform.

This study also has many strengths. To our knowledge, this is the first study that evaluated the real-life impact of behavior change with online food ordering, diet, and meal planning through a digital intervention and its impact on diabetes and HbA_1c_ levels. Using Foodsmart’s large user base, this study was able to draw real-world associations between changes in dietary habits and HbA_1c_ levels and the use of a commercial digital health platform. Furthermore, participants on the Foodsmart platform had a broad range of durations of enrollment; this allowed us to measure changes in HbA_1c_ over different lengths of time, including time spans of greater than 2 years.

### Conclusions

This study evaluated changes in self-reported HbA_1c_ levels among participants with diabetes who were using a digital nutrition intervention with personalized recipe recommendations, meal planning, food ordering, and grocery discounts and price comparisons. Future research through a randomized controlled trial will be needed to assess the causal effect of the Foodsmart platform on dietary changes and improvements in HbA_1c_ levels, the difference in cost between pharmaceutical and digital interventions, and which specific components of the dietary score are associated with a reduction in HbA_1c_ levels.

## Abbreviations

GLP-1: glucagon-like peptide 1
OR: odds ratio
SGLT2: sodium–glucose cotransporter 2
